# Rationale-Based Engineering of a Potent Long-Acting FGF21 Analog for the Treatment of Type 2 Diabetes

**DOI:** 10.1371/journal.pone.0049345

**Published:** 2012-11-27

**Authors:** Randy Hecht, Yue-Sheng Li, Jeonghoon Sun, Ed Belouski, Michael Hall, Todd Hager, Junming Yie, Wei Wang, Dwight Winters, Stephen Smith, Chris Spahr, Lei-Ting Tam, Zhongnan Shen, Shanaka Stanislaus, Narumol Chinookoswong, Yvonne Lau, Allen Sickmier, Mark Leo Michaels, Thomas Boone, Murielle M. Véniant, Jing Xu

**Affiliations:** 1 Department of Protein Sciences, Amgen Inc., Thousand Oaks, California, United States of America; 2 Departments of Metabolic Disorders, Amgen Inc., Thousand Oaks, California, United States of America; 3 Department of Pharmacokinetics and Drug Metabolism, Amgen Inc., Thousand Oaks, California, United States of America; University of Colorado Denver, United States of America

## Abstract

Fibroblast growth factor 21 (FGF21) is a promising drug candidate for the treatment of type 2 diabetes. However, the use of wild type native FGF21 is challenging due to several limitations. Among these are its short half-life, its susceptibility to *in vivo* proteolytic degradation and its propensity to *in vitro* aggregation. We here describe a rationale-based protein engineering approach to generate a potent long-acting FGF21 analog with improved resistance to proteolysis and aggregation. A recombinant Fc-FGF21 fusion protein was constructed by fusing the Fc domain of human IgG1 to the N-terminus of human mature FGF21 via a linker peptide. The Fc positioned at the N-terminus was determined to be superior to the C-terminus as the N-terminal Fc fusion retained the βKlotho binding affinity and the *in vitro* and *in vivo* potency similar to native FGF21. Two specific point mutations were introduced into FGF21. The leucine to arginine substitution at position 98 (L98R) suppressed FGF21 aggregation at high concentrations and elevated temperatures. The proline to glycine replacement at position 171 (P171G) eliminated a site-specific proteolytic cleavage of FGF21 identified in mice and cynomolgus monkeys. The derived Fc-FGF21(RG) molecule demonstrated a significantly improved circulating half-life while maintaining the *in vitro* activity similar to that of wild type protein. The half-life of Fc-FGF21(RG) was 11 h in mice and 30 h in monkeys as compared to 1-2 h for native FGF21 or Fc-FGF21 wild type. A single administration of Fc-FGF21(RG) in diabetic mice resulted in a sustained reduction in blood glucose levels and body weight gains up to 5-7 days, whereas the efficacy of FGF21 or Fc-FGF21 lasted only for 1 day. In summary, we engineered a potent and efficacious long-acting FGF21 analog with a favorable pharmaceutical property for potential clinical development.

## Introduction

Fibroblast growth factor 21 (FGF21) is a novel class of drug candidates for the treatment of metabolic disorders, including type 2 diabetes and obesity. Although FGF21 belongs to the FGF superfamily, the function of FGF21 is largely limited in metabolism ([1] Long, 2011 #387). Available pharmacological and genetic evidence support that FGF21 plays a critical role in the maintenance of glucose, lipid and energy homeostasis. Administration of FGF21 in preclinical animal models led to reductions of blood glucose, insulin, triglyceride and cholesterol levels, and a decrease in body weight [2], [3], [4], [5]. Transgenic mice overexpressing FGF21 were lean and insulin sensitive, whereas FGF21 knockout mice were mildly obese and insulin resistant [6], [7]. FGF21 regulates glucose homeostasis through a combination of multiple mechanisms. It stimulates glucose uptake in adipose tissues, suppresses glucose output in liver and preserves β-cell mass and islet function in pancreas [2], [4], [8]. Systemic administration of FGF21 improves insulin sensitivity, spares insulin secretion and ameliorates hyperinsulinemia [5], [9]. The mechanism by which FGF21 lowers plasma triglycerides is partly through inhibiting hepatic fatty acid synthesis, while increasing fatty acid oxidation. FGF21 inhibits the nuclear maturation of sterol regulatory element binding protein 1, a master lipogenic transcription factor, and thereby decreasing hepatic fatty acid and triglyceride synthesis and secretion [5], [10]. FGF21 also stimulates 5′-adenosine monophosphate activated protein kinase activity and enhances mitochondrial fatty acid oxidation [11]. The weight-loss effect of FGF21 is largely accomplished by regulating energy metabolism. Peripheral or central administration of FGF21 increases basal metabolic rate and locomotor activity without reducing food intake [5], [12], [13].

There is significant interest in developing FGF21 as a therapeutic agent to treat metabolic diseases. The human FGF21 gene encodes a 28 amino acid (aa) leading signal peptide followed by a 181 aa mature protein. Recombinant human mature FGF21 was generated and its half-life was determined to be about 1 h in rodents and monkeys [3], [4]. To achieve proper exposure and desired metabolic benefits, it was administered daily or twice daily in preclinical animal models [3], [5] and is predicted to require a frequent dosing regimen in human. This would pose a significant challenge for the use of native human FGF21 as a therapeutic molecule. Here we describe our efforts to generate a long-acting FGF21 analog with a favorable pharmaceutical property for potential clinical development. We chose to fuse FGF21 to the Fc fragment to extend its serum half-life. During the course of our studies, additional liabilities were also identified. FGF21 was found susceptible to *in vivo* proteolytic degradation and suffered a rapid loss of its *in vivo* potency and efficacy. FGF21 was also found prone to form self-association aggregates *in vitro,* posing a significant challenge for manufacturing and raising a concern for human safety as aggregates could provoke immunogenicity and cause injection site reaction. We therefore took a systematic engineering approach and generated a long-acting FGF21 analog resistant to proteolysis and aggregation. The molecular engineering of the lead molecule is described here and the efficacy characterization of the molecule in rodents and cynomolgus monkeys was published previously [14].

## Materials and Methods

### Construction of Expression Plasmids

A DNA sequence encoding the wild type human mature FGF21 polypeptide (without the leading signal peptide) was obtained by polymerase chain reaction (PCR) amplification with primers containing the nucleotide sequences corresponding to the 5′ and 3′ ends of the mature FGF21 sequence. The forward primer was 5′-AGGAGGAATAACATATGCATCCAATTCCAGATTCTTCTCC-3′ and the reverse primer5′-TAGTGAGCTCGAATTCTTAGGAAGCGTAGCTGG-3′. Restriction endonuclease sites were incorporated into the primers for directional cloning of the sequence into a proprietary expression plasmid pAMG33 (Amgen; Thousand Oaks, CA) driven by a modified inducible *lac* promoter. PCR amplication reactions followed standard protocols of molecular biology.

Fc-FGF21 fusion DNA was prepared in series of three PCR amplification reactions. In the first reaction, a pair of primers was designed to produce a sequence containing a NdeI cloning site, a Fc region of human immunoglobulin G1 (IgG1), and a linker sequence. In the second reaction, a pair of primers was designed to produce a sequence containing an overlapping portion of the linker, a portion of the FGF21 coding sequence, and an EcoRI cloning site. Finally, in the third reaction, the two flanking primers were used to link the products of the first two reactions. The product of the final reaction was digested with the restriction endonucleases NdeI and EcoRI, and then ligated to pAMG33.

Mutagenesis and truncations were achieved with primers having sequences that are homologous to DNA upstream and downstream of the codon (or codons) to be mutated or removed. The primers used in such amplification reactions also provided approximately 15 nucleotides of overlapping sequence to allow for recircularization of the amplified product, namely the entire vector including the desired changes. Amplification products were digested with the restriction endonuclease DpnI, and then transformed into competent cells. Every clone was sequenced to confirm the absence of polymerase-generated errors.

### Protein Expression and Purification

Plasmid constructs encoding various FGF21 proteins were transformed and expressed in a bacterial expression system. The expression was induced by adding IPTG and the expressed FGF21 variant protein was mainly identified in the insoluble inclusion bodies. Double-washed inclusion bodies were first solubilized in a buffer (8 M guanidine hydrochloride, 50 mM Tris, 10 mM DTT, pH 8.5) for one hour at room temperature. The solubilization mixture was then added to refold buffer (2 M urea, 0.1 M arginine, 4 mM cysteine, 4 mM cystamine dihydrochloride, pH 9.5) and mixed for 24 hours at 5°C. The refolded protein was clarified by a 4000-rpm spin in a Beckman J6B for one hour. The clarified, refolded material was dialyzed against an ion-exchange chromatography equilibration buffer. The desired protein was then purified by ion exchange chromatography column, followed by hydrophobic interaction chromatography. Peak fractions were analyzed by SDS-PAGE. The purified protein was then concentrated in Centriprep 10 (3.2 K in J6B x 1 hour). The purified protein was dialyzed in a Pierce Slide-alyzer with 10,000 MWCO against the formulation buffer consisting 10 mM potassium phosphate, 5% sorbitol at pH 8.0. Following dialysis, the protein solution was sterile filtered using a 0.2 micron filter. The identity of the protein was confirmed by analytical methods (i.e., mass spectrometry, aa sequencing, etc.). The purified proteins presented low endotoxin levels.

### Size-Exclusion Chromatography (SEC)-HPLC Analysis

Purified FGF21 variants were formulated in a buffer consisting 10 mM potassium phosphate, 5% sorbitol at pH 8.0. FGF21 variants were then concentrated to 60±10 mg/mL with an Amicon Centriprep 10, or Centricon 10 centrifugal filter device in a Beckman J6B centrifuge. The samples were spun at 3,000 RPM and incrementally checked for protein concentration via UV absorbance at 280 nm using the Nanodrop ND-1000 spectrophotometer. When the target concentration was obtained, samples were incubated at 4°C or room temperature for various amounts of time. Samples were analyzed with SEC-HPLC to monitor the formation of high molecular weight species. Experiments were performed on a Beckman HPLC system equipped with a Bio-Rad Bio-Sil SEC-250 column with 2x phosphate-buffered saline containing 2% isopropyl alcohol as the mobile phase and a flow rate at 0.6 mL/min.

### βKlotho Binding Assay

The binding of FGF21 variants to human βKlotho was tested in solution equilibrium binding assay on a BIAcore instrument as described previously [15]. Briefly, Neutravidin (Pierce Cat# 31000, Rockford, IL) was immobilized on a CM5 chip using amine coupling (reagents provided by BIAcore Inc.) with an approximate density of 5000 RU. Biotin-FGF21 was captured on the second flow cell to approximately 700 RU. The first flow cell was used as a background control. Each FGF21 variant with increased concentrations was incubated with 10 nM human βKlotho in a PBS solution containing 0.1 mg/mL BSA, 0.005% P20 for 1 hour at room temperature. The incubation mixtures were then injected over the biotin-FGF21 surface. The signal obtained with no FGF21 in the solution represents 100% βKlotho binding to the immobilized biotin-FGF21 surface. Decreased signal with increasing concentrations of a given FGF21 variant indicates that the tested FGF21 variant binds to βKlotho in solution leading to reduced βKlotho binding to the immobilized biotin-FGF21 surface on BIAcore. Data are expressed as % βKlotho binding to Biotin-FGF21 in a function of molar concentrations of the tested FGF21 variant. EC_50_ was calculated using nonlinear one-site competition model using GraphPad Prism 5.

### Elk1-Luciferase Reporter Assay

The Elk1-luciferase reporter assay was conducted in human 293T cells stably expressing human βKlotho [15] or in AM-1/D Chinese Hamster Ovary (CHO) cells stably expressing both human βKlotho and human FGFR1c (Amgen proprietary cell line derived from CHO cells). Both cell lines were also stably expressing reporter constructs encoding 5xUAS luciferase and GAL4 DNA-binding domain fused to Elk1 (GAL4-Elk1) as described previously [15]. The cDNA construct encoding human βKlotho was obtained by PCR amplification and subcloned in an expression vector pTT14. Cells were plated in 96 well plates at a density of 1×10^5^/well in a selection medium HGDMEM [Invitrogen, Carlsbad, CA] supplemented with 5% FBS, and 1× PSG. The following day, FGF21 variants were added to achieve indicated concentrations. Cells were cultured for 6 hours and lysates were collected for luciferase activity measured by Bright-Glo Luciferase Assay kit (Promega, Madison, MI). Elk1-luciferase reporter assay conducted in murine NIH/3T3 fibroblasts (ATCC, Cat # CRL-1658, Manassas, VA) were performed by transiently transfecting plasmids encoding human βKlotho and the luciferase reporter constructs (5x USA luciferase and GAL4-ELK1). Treatments and assays were carried out similarly as describe in 293T cells.

### ERK Phosphorylation Assay

The fully differentiated human primary adipocytes were ordered from Zen-Bio ([Cat# SA1006-SL, Research Triangle Park, NC). The adipocytes were derived by differentiating the pooled pre-adipocytes isolated from subcutaneous adipose tissue of healthy non-diabetic donors. After 3 hours starvation in a low glucose 1∶1 DMEM/F12 media [Invitrogen, Carlsbad, CA] supplemented with 0.3% BSA, cells were treated with increasing concentrations of FGF21 variants for 10 minutes. Cells were washed and lysates were harvested. Phosphorylated and total ERK1/2 was measured using MSD Phospho/Total ERK1/2 whole cell lysate kit (Cat# K15107D, Meso-Scale Discovery, Gaithersburg, MD) following the manufacturer's protocol. The amount of phosphor-ERK was normalized by dividing phosphor ERK signal with the total plus phosphor ERK signals.

### Animals

All mouse studies were conducted at Amgen Inc. (Thousand Oaks, CA) and were approved by the Institutional Animal Care and Use Committee (IACUC). Male C57BL/6 lean mice (Cat# 000664), *db/db* (Cat # 000697) or *ob/ob* mice (Cat # 000632) were ordered at 6–7 weeks of age from Jackson Lab (Bar Harbor, ME). Mice were maintained in rooms with a 12-h light/dark cycle, temperature between 20 and 26°C and humidity between 30 to 70%. Mice had free access to food and water. The cynomolgus monkey study was conducted at MPI Research, Inc. (Mattawan, MI) and was approved by MPI IACUC. Monkeys were single housed in stainless steel cages and were provided environmental enrichment. Monkeys were fed a certified primate diet (PMI #5048, Richmond, IN) daily in amounts appropriate for the age and size of the animals, and had ad libitum access to water. Monkeys were maintained on a 12∶12 hr light: dark cycle in rooms with temperature maintained between 18 and 28°C and humidity between 30 to 70%.

### Efficacy Studies in Diabetic Mice

After 2 weeks in house acclimation, 8–9 week old male *db/db* or *ob/ob* mice were randomized into vehicle or treatment groups using body weight and blood glucose levels as stratification criteria. Mice were then intraperitoneally (ip) administered with vehicle or FGF21 variants at indicated doses. Blood glucose levels and body weights were measured at different time points before and after compound injection. Blood samples were taken from retro-orbital sinus of *ad libitum*-fed conscious mice and blood glucose levels were measured with a One Touch Glucometer (LifeScan, Inc. Milpitas, CA).

### Pharmacokinetic and Stability Studies in Mice and Cynomolgus Monkeys

FGF21 compounds were administered to male C57BL/6 mice or cynomolgus monkeys as a single intravenous (iv) or subcutaneous (sc) injection at indicated concentrations. Blood samples were collected at indicated time points into ice-cold EDTA tubes coated with a protease inhibitor cocktail (Roche Diagnostics, Indianapolis, IN) and centrifuged at 4°C immediately. Plasma samples were divided into aliquots and stored at −80°C for later mass spectrometry analysis and ELISA assay.

### Ligand-Binding Mass Spectrometry (LBMS) Analysis

LBMS methodology has been described previously [16] and detailed MALDI-TOF and LC-ESI-MS analysis to examine *in vivo* stability of FGF21 compounds is reported elsewhere. Briefly, native FGF21 and its associated metabolites were affinity purified from plasma using anti-human FGF21 resin. Fc-FGF21 fusion proteins were affinity purified in a similar manner using anti-human Fc resin, with reduction/alkylation either before or after affinity purification. For MALDI-TOF analysis, affinity purified material was mixed with sinapinic acid solution [12 mg/mL in 33% (v/v) methanol, 0.8% (v/v) trifluoroacetic acid], transferred directly to a stainless steel target, and spectra were acquired on a Bruker Autoflex II using an optimized positive-ion linear mode method. For LC-ESI-MS analysis, affinity purified material was injected onto an ACT Ace (Aberdeen, Scotland) CN 0.3 mm X 30 cm HPLC column heated to 55°C. The column effluent was sprayed into a Thermo Finnigan (San Jose, CA) LCQ Classic ion-trap mass spectrometer. Full MS data was collected from *m/z* [400–2000]. Spectra were averaged, and then deconvoluted using the BioWorks 3.1 Software (Thermo).

### ELISA Assay

A sandwich ELISA assay optimized for measurement of the full length FGF21 moiety was developed in house. Antibody binding epitopes were identified by competitive displacement experiments using peptide fragments of native human FGF21. A murine monoclonal antibody specific for the N-terminus of human FGF21 was used as a capture antibody. A rabbit polyclonal antibody specific for the C-terminus of human FGF21 was used as a detection antibody. The assay does not recognize FGF21 fragments with a loss of 3 or more amino acids at the C-terminus. The reactivity of the antibodies to human FGF21 was confirmed by Western blot analysis. Details of the ELISA assays developed to measure various forms of FGF21 (intact or truncated) are reported elsewhere.

The ELISA assay was conducted as follows. A murine anti-human FGF21 N-terminal specific monoclonal antibody was used as a capture antibody bound onto a 96-well plate. Standards and quality controls were prepared by spiking the FGF21 analog into 100% cyno or mouse plasma. Standards, quality controls, matrix blank and unknown samples were loaded into the wells after pretreatment in assay buffer. After 2 h incubation followed by washing, a biotin-conjugated rabbit anti-human FGF21 C-terminal specific polyclonal antibody was added to the wells as the detection antibody. After washing, a streptavidin-horseradish peroxidase (HRP) conjugate was added to the wells. After a final wash step, a tetramethylbenzidine (TMB) – peroxidase substrate was added to the wells and produced a colorimetric signal measured at 450 nm with reference to 650 nm. The conversion of OD units to concentration for the unknown samples was achieved through a software-mediated comparison to a standard curve assayed on the same plate. The data underwent regression analysis using the Watson (v7.0.0.01; Thermo Fisher Scientific) data reduction package.

### Pharmacokinetic Analysis

Pharmacokinetic parameters such as area under the concentration-time curve (AUC) were estimated from FGF21 variants plasma concentration data via noncompartmental analysis using WinNonlin Professional software version 4.1e (Pharsight, Mountain View, CA). Detailed analysis was as described previously [4].

### Statistical Analysis

Data are presented as means ± SEM. Statistical comparison of the means among the groups was made using one-way ANOVA. Differences between the means of individual groups were analyzed by the post hoc Fisher's test using Statview software (SAS Institute, Inc. Cary, NC).

## Results

### Determination of an optimal fusion orientation for the maximal activity of FGF21

**Figure 1:**
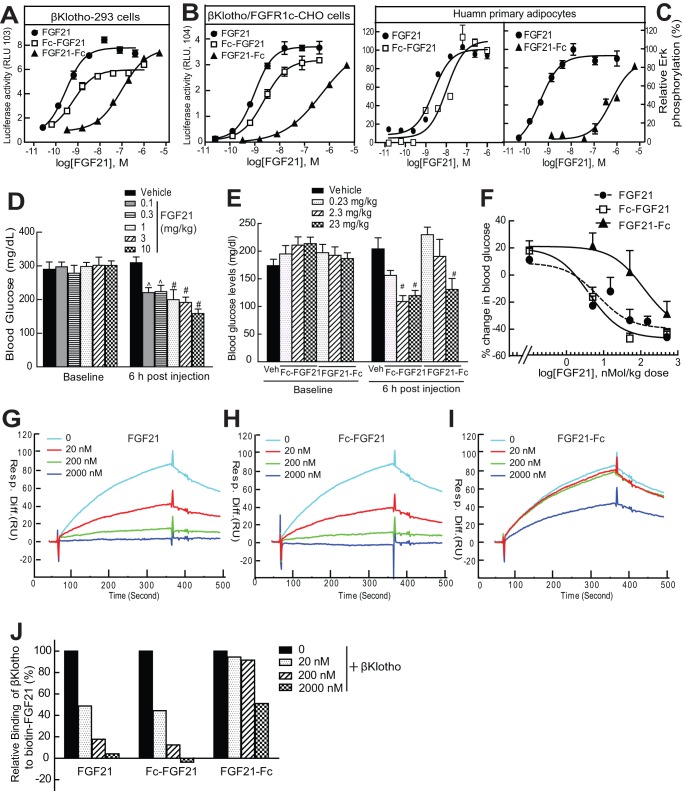
The N-terminal Fc fusion (Fc-FGF21) was superior to the C-terminal Fc fusion (FGF21-Fc) in retaining the biological activity of FGF21. (A–B) Stimulation of Elk1-luciferase reporter activity by FGF21, Fc-FGF21 and FGF21-Fc in 293T cells stably expressing human βKlotho (A) or in CHO cells stably expressing both human βKlotho and human FGFR1c (B); (C) Activation of Erk phosphorylation by FGF21, Fc-FGF21 and FGF21-Fc in human primary adipocytes. All *in vitro* data represent mean ± SEM, n = 4/concentration group. (D-F) 8-9 week old male *db/db* (C57/BL6 strain) mice were ip administered with various doses of native FGF21 (D), Fc-FGF21 or FGF21-Fc (E). Blood glucose levels were measured at baseline and 6 h after injection (D–E). % change of blood glucose levels from baseline was plotted as a function of FGF21 molar doses (F). Data are mean ± SEM, n = 9–10 animals per group, ^ p<0.01; # p<0.001 compared with vehicle. (G–J) The binding activity of FGF21 (G), Fc-FGF21 (H) and FGF21-Fc (I) to human βKlotho determined in a solution equilibrium binding assay on a BIAcore instrument. (J) Quantification of βKlotho binding to the biotinylated FGF21 immobilized on the chip. 100% βKlotho binding is the signal obtained with no FGF21 in the solution.

To extend the circulating half-life, FGF21 was fused to the Fc fragment of human IgG1 comprising the hinge region, CH2 and CH3 domains. An optimal fusion orientation was determined by generating two Fc fusion proteins by attaching the Fc to either the N-terminus (Fc-FGF21) or the C-terminus of FGF21 (FGF21-Fc) with an intervening sequence of 15 glycine and serine residues as a linker. The *in vitro* activity of the Fc fusion proteins was tested and compared with that of native FGF21 by measuring their ability to stimulate βKlotho-dependent FGF receptor (FGFR) downstream signaling. In an Elk1-dependent luciferase reporter assay conducted in 293T cells stably overexpressing human βKlotho (Figure 1A) or in CHO cells overexpressing both human βKlotho and human FGFR1c (Figure 1B), Fc-FGF21 produced approximately 80% of the maximal activity relative to native FGF21. In the same assays, FGF21-Fc was much less potent than native FGF21 or Fc-FGF21 with a dramatically right-shifted dose-response curve, although it reached the maximal activity at high concentrations (Figure 1A &1B). Similarly, in human primary adipocytes, Fc-FGF21 demonstrated a comparable potency and full agonist activity relative to native FGF21 in stimulation of Erk phosphorylation, whereas FGF21-Fc showed a drastic reduced potency by >3 orders of magnitude as indicated by a right-shifted dose-response curve (Figure 1C).

When administered in diabetic *db/db* mice (C57BL6 strain), Fc-FGF21 reduced blood glucose levels and the maximal effect was achieved at a dose of 2.3 mg/kg, whereas a similar glucose reduction required 23 mg/kg FGF21-Fc (Figure 1E). Native FGF21 also showed a potent glucose-lowering activity and its efficacy was observed at low doses (Figure 1D). The glucose-lowering dose-response curves of native FGF21 and Fc-FGF21 were nearly overlapping with a half maximal effective dose (ED50) estimated <1 nmol/kg, whereas the dose-response curve of FGF21-Fc was right-shifted and the ED50 was about 10 fold higher than that of native FGF21 or Fc-FGF21 (Figure 1F).

The βKlotho binding activity of Fc fusion proteins was also tested and compared with that of native FGF21. In a solution equilibrium binding assay, increasing concentrations of native FGF21 or Fc-FGF21 reduced free βKlotho to bind to biotinylated FGF21 immobilized on the chip surface of BIAcore instrument (Figure 1G, 1H & 1J). The binding dose-response curve of native FGF21 and Fc-FGF21 was similar. In contrast, Fc-FGF21 displayed a markedly reduced affinity to βKlotho and a modest binding was only observed at the highest tested concentration (Figure 1I &1J).

### *In vivo* stability of FGF21 in mice

**Figure 2:**
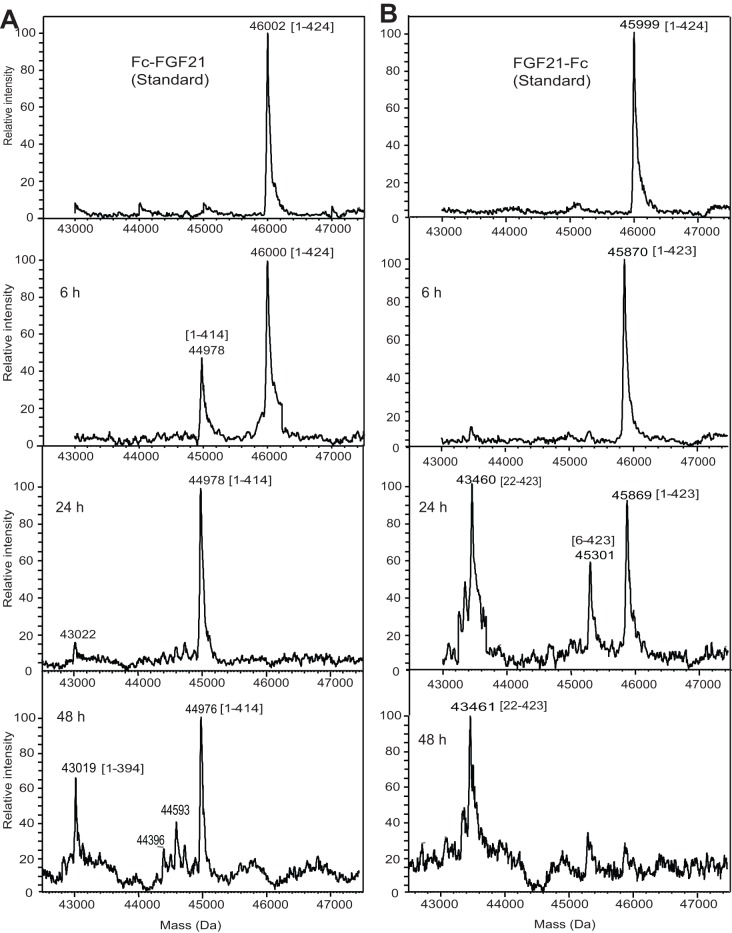
LBMS (LC-ESI-MS) analysis of Fc-FGF21 (A) or FGF21-Fc (B) in male C57BL6 mice after iv administration at 23 mg/kg. Blood samples were collected at 6, 24 and 48 h post injection and were pooled from 6 mice per time point for LBMS analysis. Top panel: standards. The predicted fragment is indicated in the bracket next to the mass peak.

The *in vivo* stability of Fc-FGF21 and FGF21-Fc was investigated in mice after i.v. administration using LBMS (LC-ESI-MS) analysis. FGF21 was rapidly cleaved in mice regardless of its fusion configurations. For Fc-FGF21, a metabolite peak (44978 Da) was detected 6 h post dose (Figure 2A). By 24 h, this metabolite became predominant and the parent (46000 Da) was no longer detectable. The mass difference between the metabolite and the parent corresponded to the mass loss of the last 10 aa residues at the C-terminus, suggesting that Fc-FGF21 was rapidly cleaved between Pro171 and Ser172 resulting in a metabolite fragment of 1–414. At 48 h, further upstream cleavages were observed. The N-terminus of Fc-FGF21 remained intact at all time points analyzed by Edman sequencing (data not shown). For FGF21-Fc, one residue at the C-terminus was rapidly missing 6 h post injection (Figure 2B). At 24 h, multiple N-terminal degradation fragments were observed and the two predominant mass peaks corresponded to the fragments of 6–423 and 22–423. By 48 h, the parent peak was minimally detected and the predominant metabolite was 22–423.

### Identification of Proteolysis-resistant Mutants

The FGF21-Fc modality was abandoned due to the reduced potency and the substantial N-terminal FGF21 degradation *in vivo.* The N-terminal Fc fusion configuration (Fc-FGF21) was chosen for further optimization to improve *in vivo* proteolysis-resistance. Mutagenesis was conducted at the aa sequence flanking the 171–172 cleavage site with an aim to identify aa substitutions that could eliminate the observed degradation while preserving the biological activity of FGF21. Single, double and triple point mutations were introduced based on sequence conservation with other species or biochemical conservation with other amino acid residues. Several single point mutations, such as G170E, P171A and P171G, were found sufficient to ablate the cleavage at 171–172 as the metabolite fragment (1–414) was no longer detectable (Table 1). The mutations did not compromise the biological activity of FGF21 (Table 1). Not all mutations introduced conferred resistance to proteolysis. For example, the S172L mutation delayed but did not prevent the cleavage at 171–172 (Table 1). It was noted that once the internal cleavage site at position 171 was eliminated, a late term carboxypeptidase-like cleavage generating 1–3 residues loss from the extreme C-terminus was initiated (Table 1).

**Table 1:** LC-ESI-MS analysis of metabolites of Fc-FGF21 variants in male C57BL6 mice following i.v. administration.

Fc-FGF21 variants	EC_50_ (nM)	Time (h)	Major observed mass (Da)	Predicted fragments	Percent of total (%)	N-terminal intact?
Fc-FGF21	4.3±0.5	0	46,002	1–424	100	―
(WT)		6	46,000	1–424	70	Yes
			44,978	1–414	30	Yes
		24	44,978	1–414	100	Yes
Fc-FGF21	6.3±0.5	0	46,068	1–424	100	―
(G170E)		6	46,078	1–424	100	Yes
		24	46,074	1–424	80	Yes
			45,761	1–421	20	Yes
Fc-FGF21	3.8±0.2	0	45,970	1–424	100	―
(P171A)		6	45,980	1–424	100	Yes
		24	45,973	1–424	70	Yes
			45,657	1–421	30	Yes
Fc-FGF21	2.7±0.5	0	45,962	1–424	100	―
(P171G)		6	45,960	1–424	100	Yes
		24	45,961	1–424	major	Yes
			45,868	1–423	minor	Yes
			45,807	1–422	minor	Yes
			45,629	1–421	minor	Yes
Fc-FGF21	5.2±0.6	0	46,022	1–424	100	―
(S172L)		6	46,027	1–424	100	Yes
		24	44,984	1–414	100	Yes

Each Fc-FGF21 variant was iv administered at 23 mg/kg into male C57BL6 mice. Blood samples were collected at 6, 24 and 48 h post injection and were pooled from 6 mice per time point for ligand binding LC-ESI-MS analysis. Full-length Fc-FGF21 contains 424 aa residues. * Poor resolutions for the quantification of each mass peak; 0 h time point represents reduced standard.

### Identification of Aggregation-resistant Mutants

**Figure 3:**
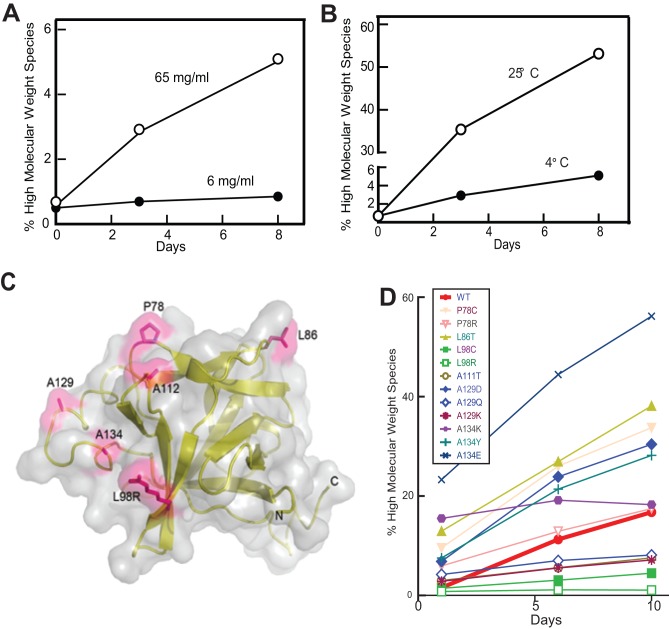
Identification of aggregation-resistant FGF21 mutants. (A–B) FGF21 aggregation was concentration-(A), temperature- (B), and time-dependent (A&B). The % of high molecular weight species over the total was obtained from size exclusion chromatography (SEC) analysis. (C) Homology model of FGF21 (residues 14–155) with the mutated residues shown in magenta. The leucine to arginine mutation is shown for residue 98 (magenta) and all other mutation sites were shown as native sequence. The FGF21 homology model was prepared using Modeler (Fiser and Sali) in Discovery studio 3.1 (Accelrys) with FGF19 (PDB code: 2P23) used as a template. Image was created using PyMol (Schrödinger, LLC). (D) The aggregation rate of FGF21 mutants. Samples were concentrated at 60±10 mg/ml and stored at 4°C for 1, 6 and 10 days before SEC analysis.

Native FGF21 was also found to have a propensity to form aggregates. The rate of aggregation increased with increasing concentrations, temperatures or times (Figure 3A & 3B). To identify aa substitutions that could reduce FGF21 aggregation without compromising its biological activity, a broad aa substitution was carried out using a computational protein engineering approach. A hypothesis was proposed that FGF21 aggregation is triggered by van der Waals interactions between surface-exposed hydrophobic residues in a hydrophilic water-based solvent environment. As there were no known X-ray or NMR structures of FGF21, a 3D homology model of FGF21 was built and was used to identify surface-exposed hydrophobic residues (Figure 3C). The model was prepared using FGF19 as a template, since FGF19 is the most closely related protein to FGF21 and has a high resolution X-ray crystal structure available. In silico point mutations were executed on the identified hydrophobic residues by replacing the given residue with 19 other aa residues. Stabilizing mutations that introduced improved hydrophilic/ionic characters were selected. The mutants having the biological activity similar to FGF21 were selected for aggregation analysis at high concentrations (Table 2 & Figure 3D). The FGF21(L98R) mutant displayed the least aggregate formation rate of any mutant tested.

**Table 2:** In vitro activity of FGF21 variants.

FGF21 variants	EC_50_ (nM)
WT	0.44±0.05
P78C	0.36±0.04
P78R	0.16±0.03
L86T	0.30±0.04
L98C	0.32±0.06
L98R	0.23±0.06
A111T	0.64±0.07
A129D	1.25±0.12
A129Q	0.83±0.08
A129K	0.42±0.04
A134K	1.18±0.13
A134Y	0.99±0.09
A134E	2.81±0.13

293T cells stably expressing human βKlotho along with luciferase reporter constructs were treated with FGF21 variants for 6 hours and lysates were collected to measure luciferase activity.

### Generation of Fc-FGF21(RG) mutant and determination of its in vitro activity

**Figure 4:**
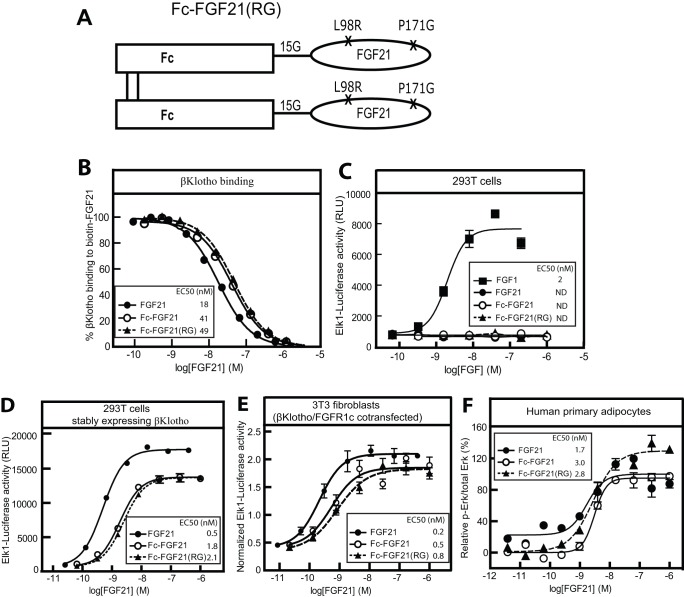
In vitro activity of Fc-FGF21(RG) relative to native FGF21 and Fc-FGF21 wild type. (A) Structural diagram of Fc-FGF21(RG), an E coli-expressed homodimeric Fc-FGF21 fusion protein containing 2 engineered point mutations at positions 98 and 171. 15G indicates the 15 aa polyglycine and polyserine linker. (B) The binding activity of FGF21, Fc-FGF21 and Fc-FGF21(RG) to human βKlotho determined in a solution equilibrium binding assay on a BIAcore instrument. (C) Elk1-luciferase reporter activity in 293T cells treated with FGF1, FGF21, Fc-FGF21 and Fc-FGF21(RG). The cells were stably expressing reporter constructs but no βKlotho. (D) Stimulation of Elk1-luciferase reporter activity by FGF21, Fc-FGF21 and Fc-FGF21(RG) in 293T cells stably expressing human βKlotho along with luciferase reporter constructs. (E) Stimulation of Elk1-luciferase reporter activity by FGF21, Fc-FGF21 and Fc-FGF21(RG) in mouse NIH/3T3 fibroblast cells transiently co-transfected with human βKlotho and human FGFR1c. (F) Stimulation of Erk phosphorylation by FGF21, Fc-FGF21 and Fc-FGF21(RG) in human primary adipocytes. All data represent mean ± SEM, n = 4/concentration group. ND: not detectable. FGF21, closed circle; Fc-FGF21: opened circle; Fc-FGF21(RG): closed triangular and dash line; FGF1: closed square.

An Fc-FGF21 fusion protein containing 2 point mutations, the aggregation-resistant mutation L98R and the proteolysis-resistant mutation P171G, was generated. The structural diagram of the Fc-FGF21(RG) was shown in Figure 4A.The *in vitro* activity of Fc-FGF21(RG) was tested and compared with that of native FGF21 or Fc-FGF21 wild type. In a solution equilibrium binding assay, Fc-FGF21(RG) bound to βKlotho with a binding EC50 similar to that of Fc-FGF21 wild type and close to that of native FGF21 (Figure 4B). At the absence of βKlotho, Fc-FGF21(RG) had no activity in an Elk1-luciferase reporter assay similarly as FGF21 and Fc-FGF21, whereas the classical growth factor FGF1 was active (Figure 4C). At the presence of βKlotho, Fc-FGF21(RG) induced Elk-luciferase reporter activity or Erk phosphorylation in various cell-based assays (Figure 4D-4F). The potency and efficacy of Fc-FGF21(RG) was comparable to that of Fc-FGF21 wild type, suggesting the 2 mutations did not compromise the biological activity of FGF21. However, compared with native FGF21, both Fc fusions showed a submaximal activity in cells overexpressing βKlotho and/or FGFR1c, whereas full agonist activity was observed in primary adipocytes, a physiologically relevant cell system with normal expression levels of βKlotho and FGFRs.

### Fc-FGF21(RG) was resistant to aggregation

**Figure 5:**
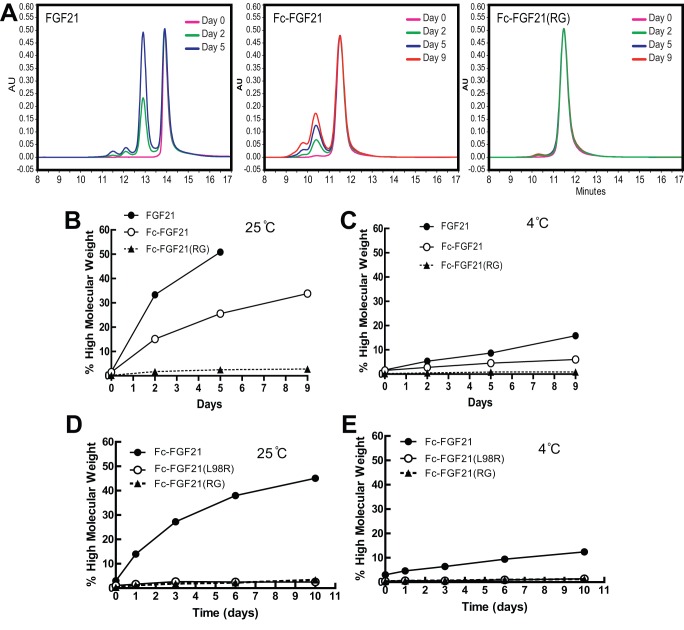
Fc-FGF21(RG) was resistant to aggregation. (A) Size exclusion chromatography (SEC) of native FGF21, Fc-FGF21 wild type and Fc-FGF21(RG) concentrated at 65 mg/ml stored at 25°C for various days. (B-C) Aggregation rate of FGF21 (closed circle), Fc-FGF21 (opened circle) or Fc-FGF21(RG) (closed triangular) at 65 mg/ml stored at either 25°C (B) or 4°C (C). (D–E) Aggregation rate of Fc-FGF21 (closed circle), Fc-FGF21(L98R) (opened circle) or Fc-FGF21(RG) (closed triangular) at 65 mg/ml stored at either 25°C (D) or 4°C (E).

To confirm Fc-FGF21(RG) inherits aggregation-resistance by harboring L98R mutation, SEC analyses was conducted for Fc-FGF21(RG) in comparison with FGF21 and Fc-FGF21. All compounds were formulated in a buffer consisting 10 mM potassium phosphate, 5% sorbitol, pH 8.0 and concentrated at 65 mg/ml at 25°C. At time 0, a single peak was observed for each compound suggestive of monomeric form. By day 2, additional peaks with shorter retention times were observed for native FGF21 and Fc-FGF21 but was not for Fc-FGF21(RG) (Figure 5A). The intensity of the aggregates increased with time and by day 5, the high molecular aggregates accounted for 50% and 25% of total native FGF21 and Fc-FGF21, respectively (Figure 5A & 5B). Compared with native FGF21, Fc-FGF21 had 50% reduced aggregation rate at both 25°C and 4°C, whereas Fc-FGF21(RG) demonstrated resistance to aggregation over a period of 9 days and the amount of aggregates remained below 3% at all tested conditions (Figure 5A–5C). A further study confirmed that the L98R mutation conferred the aggregation resistance to Fc-FGF21 fusion proteins (Figure 5D &5E).

### Fc-FGF21(RG) was resistant to proteolysis in cynomolgus monkeys

**Figure 6:**
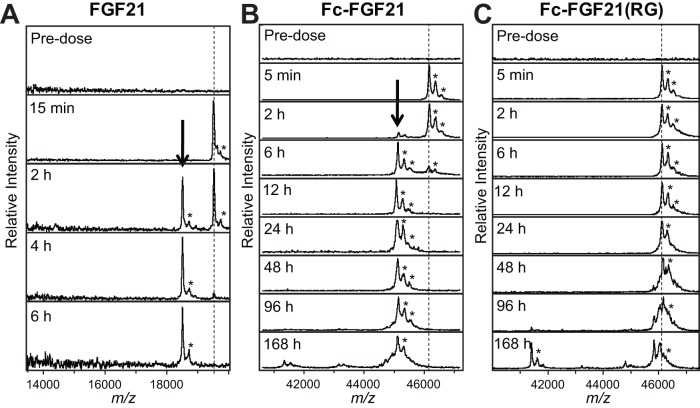
Fc-FGF21(RG) was resistant to degradation in cynomolgus monkeys. LBMS (MALDI-TOF) analysis of native FGF21 (A), Fc-FGF21 (B) or Fc-FGF21(RG) (C) in male cynomolgus monkeys. FGF21 (10 mg/kg) or Fc-FGF21 variants (23.5 mg/kg) were iv administered in cynomolgus monkeys and blood samples were collected at indicated time points. The mass positions of the parent constructs are indicated by vertical hashed lines. The peak marked with a heavy arrow corresponds to the primary metabolite of FGF21 or Fc-FGF21, which is absent in the Fc-FGF21(RG). Peaks marked with asterisks in all of the spectra correspond to sinapinic acid adducts of primary peaks and are an artifact of the ionization process of MALDI. The MALDI-TOF mass spectra are normalized to the most intense peak in the plotted *m/z* range.

To investigate whether Fc-FGF21(RG) is proteolysis-resistant in a higher species, the *in vivo* stability of Fc-FGF21(RG) was determined in cynomolgus monkeys in comparison with native FGF21 and Fc-FGF21. LBMS (MALDI-TOF) analysis confirmed that the same proteolytic cleavage at the site between Pro171 and Ser172 that we had observed in mice was detected in cynomolgus monkeys (Figure 6A & 6B). The cleavage occurred for both native FGF21 and Fc-FGF21, suggesting that the wild type FGF21 sequence carried the susceptibility to this degradation. Native FGF21 was 100% cleaved 4 hr post dose (Figure 6A) and both the parent and the clipped N-terminal fragments were not detected beyond 6 h time point (data not shown). Fc-FGF21 was 100% cleaved 6 h post dose and the clipped N-terminal fragment was remarkably stable and remained detectable for 168 h (Figure 6B). In contrast, Fc-FGF21(RG) showed a resistance to the cleavage at 171–172 site over 168 h post injection (Figure 6C). However, late-term metabolic events at the extreme C-terminus of Fc-FGF21(RG) were observed in monkeys (Figure 6C) similarly to what was observed in mice (Table 1).

### Fc-FGF21(RG) demonstrated improved pharmacokinetics

**Figure 7:**
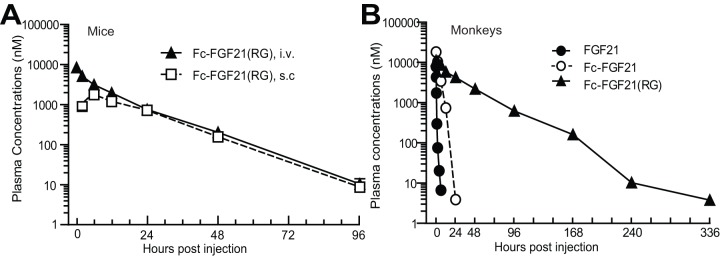
Fc-FGF21(RG) exhibited improved pharmacokinetics in mice and cynomolgus monkeys. (A) Plasma concentrations vs. time profile of Fc-FGF21(RG) following iv (closed triangular) or sc (opened square) administration at 20 mg/kg in male C57BL6 mice. (B) Plasma concentrations vs. time profile of native FGF21 (closed circle), Fc-FGF21 (opened circle) or Fc-FGF21(RG) (closed triangular) following iv administration in male cynomolgus monkeys. FGF21: 10 mg/kg; both Fc compounds: 23.5 mg/kg.

An in-house ELISA assay was developed and optimized for an accurate measurement of the active full-length FGF21 moiety (aa 1–181). The assay does not recognize the inactive species, characterized as those fragments with a loss of 3 or more aa at the C-terminus of FGF21 [15]. The pharmacokinetics (PK) of Fc-FGF21(RG) was determined in mice following iv or sc administration (Figure 7A). The terminal half-life of Fc-FGF21(RG) in mice was 11.2 h and 12 h following iv and sc administration, respectively. The bioavailability of Fc-FGF21(RG) was 52.5% in mice. Detailed pharmacokinetic parameters are listed in Table S1.

In male cynomolgus monkeys following iv administration of FGF21, Fc-FGF21 and Fc-FGF21(RG) at approximately molar equivalent doses, a linear PK was observed for all tested FGF21 variants (Figure 7B). The terminal half-life of FGF21, Fc-FGF21 and Fc-FGF21(RG) was determined to be 1.1 h, 1.8 h, and 30 h, respectively, using the intact FGF21 ELISA assay described above. Data demonstrated that the full length FGF21 was detectable up to 336 h in monkeys receiving Fc-FGF21(RG) while quickly declined in monkeys receiving native FGF21 or Fc-FGF21 (Figure 7B). The PK of FGF21 variants was consistent with their stability in monkeys.

To confirm the superior PK of Fc-FGF21(RG) was attributed to P171G mutation, a separate ELISA assay was also developed by employing an antibody pair specific to the extreme N-terminal sequence and the epitope of 160–164. Using this assay, no difference between the PK profile of Fc-FGF21 and Fc-FGF21(RG) was observed (data not shown), suggesting that the P171G mutation at a site downstream of 160–164 contributed to the improved PK, whereas the L98R mutation played a minimal part on *in vivo* PK.

### Fc-FGF21(RG) prolonged in vivo efficacy

**Figure 8:**
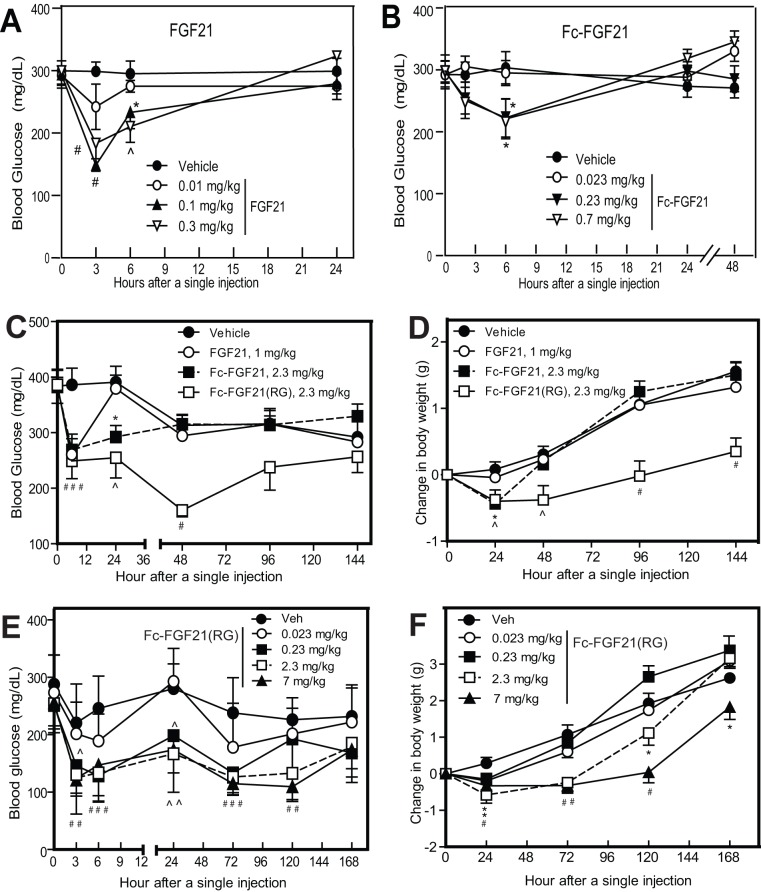
Fc-FGF21(RG) had a prolonged efficacy in *db/db* or *ob/ob* mice. (A-B) 8-9 week old male *db/db* mice were ip administered with FGF21 (A) or Fc-FGF21 (B) at various molar equivalent doses. The molar equal dose is defined as an equivalent molar dose of FGF21 as a unit. Blood glucose levels were measure at the indicated time points. (C–D) Effect of vehicle (closed circle), FGF21 (opened circle), Fc-FGF21(closed square) or Fc-FGF21(RG) (opened squared) on blood glucose levels (C) and body weight gains (D) following a single injection at a molar equivalent dose in *db/db* mice. Blood glucose levels and body weight were measured over a course of 144 h. (E–F) Dose-response and time course effects of Fc-FGF21(RG) on blood glucose levels (E) and body weight gains (F) in *ob/ob* mice. 8–9 week old male *ob/ob* mice were ip administered with various doses of Fc-FGF21(RG). Blood glucose levels and body weight were measured over a course of 168 h. All data are mean ± SEM, n = 9–10 animals per group, * p<0.05, ^ p<0.01, # p<0.001 compared with vehicle at each time point.

Native FGF21 or Fc-FGF21 was administered in diabetic *db/db* mice at various molar equivalent doses and blood glucose levels were measured at different time points following a single injection. The molar equivalence was based on FGF21 moiety. Although both molecules dose-dependently reduced blood glucose levels with a similar potency and efficacy, both molecules showed a short duration of glucose-lowering effect and by 24 hr, blood glucose levels returned to baseline (Figure 8A & 8B). In contrast, Fc-FGF21(RG) produced a prolonged blood glucose reduction and inhibition of body weight gain when administered at a molar equivalent dose to that of native FGF21 or Fc-FGF21 (Figure 8C & 8D). The effect of Fc-FGF21(RG) persisted >48 h for blood glucose reduction and >144 h for body weight gain inhibition, whereas the effects of native FGF21 or Fc-FGF21 lasted ≤24 h. Administration of Fc-FGF21(RG) also caused sustained reductions in blood glucose levels and body weight gains in *ob/ob* mice (Figure 8E & 8F). Both efficacy and duration of actions were dose-dependent.

## Discussion

Conjugation of a human IgG Fc variant to therapeutic proteins has been used to produce a number of successful commercial products. Here we describe an engineering strategy to generate a potent and efficacious Fc-fused FGF21 analog as a potential therapeutic agent to treat human type 2 diabetes. The wild type human FGF21 has several liabilities that limit its use as a therapeutic molecule. It has a short half-life necessitating a frequent dosing regimen. We also observed its other liabilities, including its susceptibility to *in vivo* degradation and its propensity to *in vitro* aggregation. A systematic rationale engineering approach was therefore carried out. Two point mutations (L98R, P171G) were identified that offered FGF21 resistance to aggregation and *in vivo* proteolysis, respectively, and were then incorporated into the N-terminally Fc fused FGF21. The derived Fc-FGF21(RG) variant demonstrated an improved aggregation- and proteolysis-resistance, a superior *in vivo* pharmacokinetics and pharmacodynamics while preserving *in vitro* activity similar to that of wild type human FGF21.

Native human FGF21 has a molecular weight of about 20 kD and exhibits a short serum half-life in rodents and monkeys analyzed by sandwich immunoassays [3], [5]. In the current study, we further confirmed that FGF21 was rapidly eliminated from the circulation not only by ELISA assay but also LBMS analysis. Both the parent and the clipped N-terminal fragments of native FGF21 were not detected beyond 6 h post dose in monkeys. Kidney filtration is likely one of the major routes for FGF21 elimination from the circulation. Evidence to support this hypothesis include human observations that serum FGF21 levels were markedly elevated in patients with severely impaired renal functions and reduced glomerular filtration rates [17], [18]. Approaches to retard kidney clearance include conjugating the protein of interest to a large carrier, such as chemically conjugating to a biocompatible polymer, or genetically fused with a naturally occurring protein presenting a long serum half-life. We chose to attach FGF21 to the Fc fragment of IgG1 isotype for several reasons. Fusion with Fc would increase the molecular weight of FGF21 from a monomer of 19.5 kD to a dimer of 92 kD, exceeding the kidney filtration cutoff of 50 kD. Fusion with Fc also would harbor the well-documented half-life extension mechanism through FcRn-mediated recyclying [19]. Additional benefits from Fc fusion also include efficient expression in bacterial host cells, reduced cost of goods and increased solubility.

The Fc fragment could be fused at either the N- or C-terminus of the gene of interest. FGF21 interacts with a receptor complex composed of a co-receptor βKlotho and a signaling receptor FGFR isoform [20], [21], [22]. Although the crystal structure of FGF21 interaction with the receptor complex has not been resolved, domain deletion studies indicated that both termini of FGF21 are critical for its biological activity and play a differential role in receptor interaction and activation [14]. The N–terminus of FGF21 is involved in FGFR interaction and is crucial for transmitting signaling activity, whereas the C-terminus of FGF21 is essential for binding to βKlotho, a prerequisite step for FGFR activation [15], [23]. In the current study, when the Fc was fused to the C-terminus of FGF21, the derived fusion protein showed a dramatically reduced binding activity to βKlotho, a decreased potency in eliciting FGFR signaling and in producing glucose-lowering effect. The Fc at the C-terminus may have constrained an optimal interaction of FGF21 with βKlotho. On the other hand, when the Fc was fused to the N-terminus of FGF21, the derived N-terminally Fc fused FGF21 showed partial agonist activity in cells overexpressing βKlotho or βKlotho/FGFR1c. The partial agonist effect is likely an *in vitro* artifact generated by coreceptor/receptor overexpression and reconstitution. The ratio of βKlotho and FGFR or the reconstituted receptor complex may not be optimal for Fc-FGF21 to induce signaling. Alternatively, the N-terminally Fc fused FGF21 is indeed a partial agonist as a result of the steric hindrance imposed by Fc. Nevertheless, the partial activity appeared mild and was not evident in primary cells or *in vivo*. Therefore, the N-terminal Fc fusion is considered superior to the C-terminal Fc fusion and is a choice of optimal fusion configuration.

We here also report a combination strategy to circumvent rapid *in vivo* proteolysis in conjugation with reducing fast clearance is necessary for half-life extension of FGF21. We observed that FGF21 is vulnerable to *in vivo* degradation at both termini. The susceptible degradation sites include positions 5–6, 21–22 at the N-terminus and 171–172 at the C-terminus. The Fc at the fusion appeared to protect the portion of the FGF21 sequence that is adjacent to the Fc sequence from degradation. When the Fc was positioned at the N-terminus, the N-terminal FGF21 was protected from degradation while the C-terminal region remained vulnerable to proteolytic attack. The homology model based on FGF19 structure predicted that FGF21 exhibits a core structure flanked by structurally disordered N- and C-terminal residues (Data not shown). Lack of rigidity and bulk structure may render the terminal domain susceptible to most proteases. For Fc-FGF21, a particular susceptible degradation site was identified located at the C-terminus between Pro171 and Ser 172. The proteolysis led to a rapid truncation of 10 aa residues at the C-terminus, which abolished the ability of FGF21 to bind to βKlotho and terminated its biological activity *in vivo* (Data not shown). A specific protease conserved between species may be involved. Site-specific mutations including P171G substitution may have eradicated the consensus recognition sequence of a particular protease and therefore eliminated the observed degradation at this site. Inhibition of *in vivo* proteolysis appeared particularly important for Fc-FGF21 fusion protein relative to native FGF21 as the degradation rate became predominant in determining the *in vivo* biological activity of Fc-FGF21 when the clearance rate was decreased and serum residence time was increased as a result of Fc conjugation.

It is interesting to note that FGF23, a close family member of FGF21, was also sensitive to C-terminal proteolysis at Arg179 and Ser180 [24]. Human mutations with either enhanced or reduced FGF23 C-terminal cleavage have been associated with severe pathogenesis in calcium and phosphate metabolism [24], [25], [26]. Alignment of the sequence between the cleavage site of FGF21 and FGF23 revealed no homology, suggesting distinct proteases may be involved. The clinical relevance of FGF21 proteolysis requires further elucidations and the identification of the protease involved in FGF21 cleavage may yield a new interrogation strategy.

FGF21 showed a tendency to form aggregates, predominantly dimeric and oligomeric self-associations. The aggregation is undesirable for a biotherapeutics as protein aggregates could induce adverse effects and are linked to immunogenicity in human. Although the molecular mechanism of FGF21 aggregation remains unknown, we hypothesized that the aggregation was triggered by favorable protein: protein interactions between surface-exposed hydrophobic residues. A location of a lysine residue at position 98 was found to be critically involved in FGF21 aggregation. A single arginine replacement suppressed FGF21 *in vitro* aggregation observed at high concentrations or at elevated temperatures over time. Since the L98R mutation showed improved hydrophilic and/or ionic characteristics, it may have eliminated the hydrophobic surface interactions between FGF21 at the particular locus and attest our hypothesis.

Computer algorithms predicted the two mutations we introduced to FGF21 were not likely to break immune tolerance or increase immunogenicity (Data not shown). The two mutations also did not compromise FGF21 activity. Compared to wild type Fc-FGF21 fusion protein, the Fc-FGF21(RG) displayed a similar binding activity to βKlotho and a comparable ability to elicit FGFR downstream signaling. The *in vitro* activity of Fc-FGF21(RG) was also close to that of native human FGF21, particularly when tested in human primary adipocytes. The pharmacokinetics of Fc-FGF21(RG) was significantly improved as result of both Fc fusion and resistance to *in vivo* proteolysis. The half-life of Fc-FGF21(RG) was 11 h in mice and 30 h in monkeys, whereas the half-life of native FGF21 or Fc-FGF21 was 1–2 h in mice and monkeys using an ELISA assay measuring full-length bioactive FGF21. A single administration of Fc-FGF21(RG) in diabetic mice resulted in a sustained reduction in blood glucose levels and inhibition of body weight gains over 5–7 days, whereas the effect of native FGF21 or Fc-FGF21 only lasted for 1–2 days. The efficacy of Fc-FGF21(RG) administered once a week was comparable to or greater than to that of native FGF21 administered once or twice a day tested in rodents and cynomolgus monkeys [14]. In summary, we demonstrated that Fc-FGF21(RG) is a potent and efficacious long-acting FGF21 analog with an improved pharmaceutical properties. Its pharmacokinetic profile supports once a week dosing regimen.

## Supporting Information

Table S1 **Pharmacokinetic parameters of FGF21 variants following iv or sc administration to male C57BL6 mice or cynomolgus monkeys.**
(DOCX)
