# Supplementary material for: Rationale-Based Engineering of a Potent Long-Acting FGF21 Analog for the Treatment of Type 2 Diabetes
Source: PLoS One. 2012 Nov 27;7(11):e49345. doi: 10.1371/journal.pone.0049345 (PMC3507880; doi:10.1371/journal.pone.0049345)
Supplement: Table S1 — Pharmacokinetic parameters of FGF21 variants following iv or sc administration to male C57BL6 mice or cynomolgus monkeys. (DOCX) [file pone.0049345.s001.docx]

Supplemental Table 1. Pharmacokinetic parameters of FGF21 variants following iv or sc administration to male C57BL6 mice or cynomolgus monkeys

| Pharmacokinetic  Parameters | Cynomolgus monkeys |  |  | C57BL6 mice |  |
| --- | --- | --- | --- | --- | --- |
|  | FGF21 | Fc-FGF21 | Fc-FGF21(RG) | Fc-FGF21(RG) | Fc-FGF21(RG) |
|  | 10mg/kg, iv | 23.5mg/kg, iv | 23.5mg/kg, iv | 20mg/kg, iv | 20mg/kg, sc |
| t_max_ (hr) | NA | NA | NA | NA | 6.0 |
| C_0_ or C_max_ (μM) | 10.5 | 18.6 | 11.1 | 8.6 | 1.8 |
| AUC_0-inf_ (μM•hr) | 3.14 | 65.6 | 325 | 73.4 | 38.5 |
| t_1/2,z_ (hr) | 1.14 | 1.80 | 30.2 | 11.2 | 12.0 |
| CL (mL/hr/kg) | 159 | 7.96 | 1.60 | 6.04 | 11.6 |
| V_0_ (mL/kg) | 47.6 | 28.0 | 46.6 | 51.5 | NA |
| V_ss_ (mL/kg) | 69.5 | 27.7 | 60.2 | 83.9 | NA |
| F (%) | NA | NA | NA | NA | 52.5 |

NA: Not Applicable

C0 = extrapolated plasma concentration at time zero

Cmax = maximum plasma concentration after SC administration

AUC0-inf = area under the concentration-time curve from zero to infinity

t1/2,z = half-life associated with the terminal phase

CL = clearance rate

V0 = initial volume of distribution

Vss = volume of distribution at steady state

F= Bioavailability relative to mean AUC0-inf of iv dose group
